# Group A *Streptococcus* exploits human plasminogen for bacterial translocation across epithelial barrier via tricellular tight junctions

**DOI:** 10.1038/srep20069

**Published:** 2016-01-29

**Authors:** Tomoko Sumitomo, Masanobu Nakata, Miharu Higashino, Masaya Yamaguchi, Shigetada Kawabata

**Affiliations:** 1 Department of Oral and Molecular Microbiology, Osaka University Graduate School of Dentistry, 1-8, Yamadaoka, Suita, Osaka, 565-0871, Japan

## Abstract

Group A *Streptococcus* (GAS) is a human-specific pathogen responsible for local suppurative and life-threatening invasive systemic diseases. Interaction of GAS with human plasminogen (PLG) is a salient characteristic for promoting their systemic dissemination. In the present study, a serotype M28 strain was found predominantly localized in tricellular tight junctions of epithelial cells cultured in the presence of PLG. Several lines of evidence indicated that interaction of PLG with tricellulin, a major component of tricellular tight junctions, is crucial for bacterial localization. A site-directed mutagenesis approach revealed that lysine residues at positions 217 and 252 within the extracellular loop of tricellulin play important roles in PLG-binding activity. Additionally, we demonstrated that PLG functions as a molecular bridge between tricellulin and streptococcal surface enolase (SEN). The wild type strain efficiently translocated across the epithelial monolayer, accompanied by cleavage of transmembrane junctional proteins. In contrast, amino acid substitutions in the PLG-binding motif of SEN markedly compromised those activities. Notably, the interaction of PLG with SEN was dependent on PLG species specificity, which influenced the efficiency of bacterial penetration. Our findings provide insight into the mechanism by which GAS exploits host PLG for acceleration of bacterial invasion into deeper tissues via tricellular tight junctions.

*Streptococcus pyogenes* (Group A *Streptococcus*, GAS) is a human-restricted pathogen that causes a wide variety of human diseases ranging from commonly mild superficial infections of the pharynx and skin to life-threatening invasive diseases such as necrotizing fasciitis^1^,^2^. GAS organisms are also responsible for autoimmune sequelae, such as rheumatic fever and glomerulonephritis, which are serious health problems in developing countries^3^. GAS colonization in the pharynx is considered to be fundamental for development of these sequelae and invasive diseases, albeit pharyngeal epithelial cells function as a physical barrier to protect underlying sterile tissue.

Cell-cell and cell-matrix junctional adhesions are essential for maintenance of polarized epithelium, which plays a significant role in resistance to infection. Tight junctions (TJs) located at the most apical site of lateral membranes seal the intercellular space between adjacent cells, forming a primary barrier against most human pathogens^4^. In particular, TJs between adjacent cells are known as bicellular tight junctions (bTJs), while tricellular tight junctions (tTJs) are observed at the point of contact of three epithelial cells. bTJs converge to tTJs, thus forming a vertically orientated triple pair strand structure, namely a central tube^5^,^6^. The central tube is assumed to be a vulnerable site of the paracellular barrier, thus tTJs may provide an entry portal for human pathogens^7^,^8^. In fact, a recent report indicated that *Shigella*-containing pseudopodia target tricellulin, a major component of the tTJs, for dissemination into neighboring cells in *Shigella* infections^9^. In the process of investigating the initial steps of GAS infection, we observed that some GAS strains have a tendency to translocate through cellular contacts where the corners of three or four polygonal epithelial cells meet, which was accompanied by degradation of junctional proteins^10^. Although the mechanism of such tricellular localization has yet to be elucidated, specific host molecules related to the tTJ structure have been suggested to mediate the process.

Multiple species of bacteria are able to sequester host plasminogen (PLG) and have been implicated in the pathogenesis of invasive diseases^11^. Indeed, several GAS surface proteins act as PLG receptors and play crucial roles in the initial stage of severe invasive GAS infection^12^. PLG is a glycoprotein abundant in plasma and extracellular fluids, such as saliva, and possesses seven structural domains; an N-terminus activation peptide, five kringle domains, and a C-terminus serine protease region. Interactions of PLG with surface-exposed GAS receptors are mediated by lysine-binding sites within the kringle domains. Interestingly, Pancholi *et al.* reported that PLG is a determinant for pericellular invasion of GAS into human pharyngeal cells^13^. However, the specific receptors on the host cell surface involved in this process have yet to be identified. On the basis of their report and our previous findings, we postulated that a PLG-mediated interplay between GAS and tTJs components is involved in the paracellular tropism of bacterial tissue invasion.

The present study was undertaken to clarify the mechanism by which GAS is predominantly localized in paracellular junctions. Our findings indicate that PLG functions as a molecular bridge between tricellulin and streptococcal surface enolase (SEN), the principal PLG-binding protein of GAS. In addition, data in the present study imply that GAS exploits the PLG-binding properties of tricellulin to translocate via tTJs of the epithelial barrier.

## Results

### PLG enhances bacterial localization in tTJs

**Figure 1:**
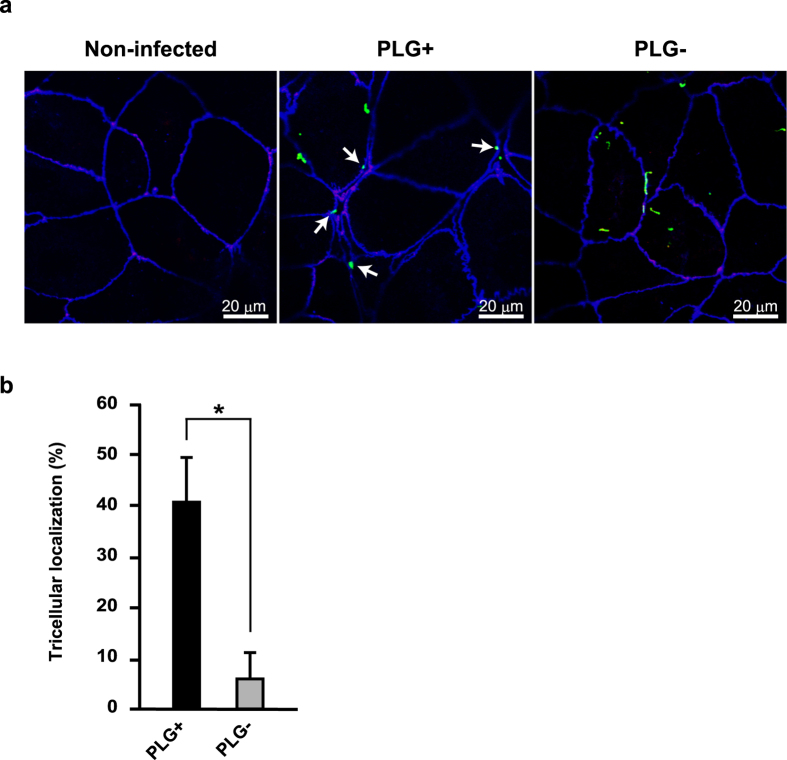
PLG is associated with bacterial localization in tTJs. (**a**) Caco-2 cells were infected with an EGFP-expressing GAS NIH35 strain as green images at an MOI of 10 for 2 h in the presence or absence of 2 μM human PLG. Tricellulin was labeled with anti-tricellulin and Alexa Fluor 594-conjugated antibodies as red images, whereas ZO-1 was labeled with anti-ZO-1 and Alexa Fluor 647-conjugated antibodies as blue images. GAS-infected cells were analyzed using a confocal laser-microscope. White arrows indicate bacterial association with tricellulin. Data shown are representatives of at least three separate experiments. (**b**) Bacterial localization in tTJs was assessed as described in the Methods section. Data obtained from ten fields of view (x630) are presented as the mean ± S.D. *P < 0.01.

As a first step to test involvement of PLG in the transmigration of GAS via an epithelial paracellular route, Caco-2 cells were infected with an EGFP-expressing NIH35 strain in the presence or absence of human PLG, and bacterial localization was analyzed using a confocal microscopy (Fig. 1a). Caco-2 cells express typical epithelial markers of differentiation and are particularly useful for studying pathogen-host interactions^14^,^15^,^16^. Since strain NIH35, a clinical M28 isolate from a patient with an invasive GAS infection, has been shown to preferentially penetrate the epithelial barrier through a paracellular route, we used that strain throughout this study^10^,^17^. Although PLG had no effect on bacterial adherence to Caco-2 cells (Supplementary Fig. 1), GAS was prone to be localized at tTJs labeled with anti-tricellulin. In contrast, the tendency of GAS localization was less prominent in the absence of PLG. Quantitative analysis also revealed that bacterial localization at tricellular contacts was significantly decreased in the absence of PLG (Fig. 1b). Based on these results, we speculated that PLG is a critical host factor for bacterial localization in tricellular contact regions.

### Tricellulin is a determinant for tricellular localization of GAS

**Figure 2:**
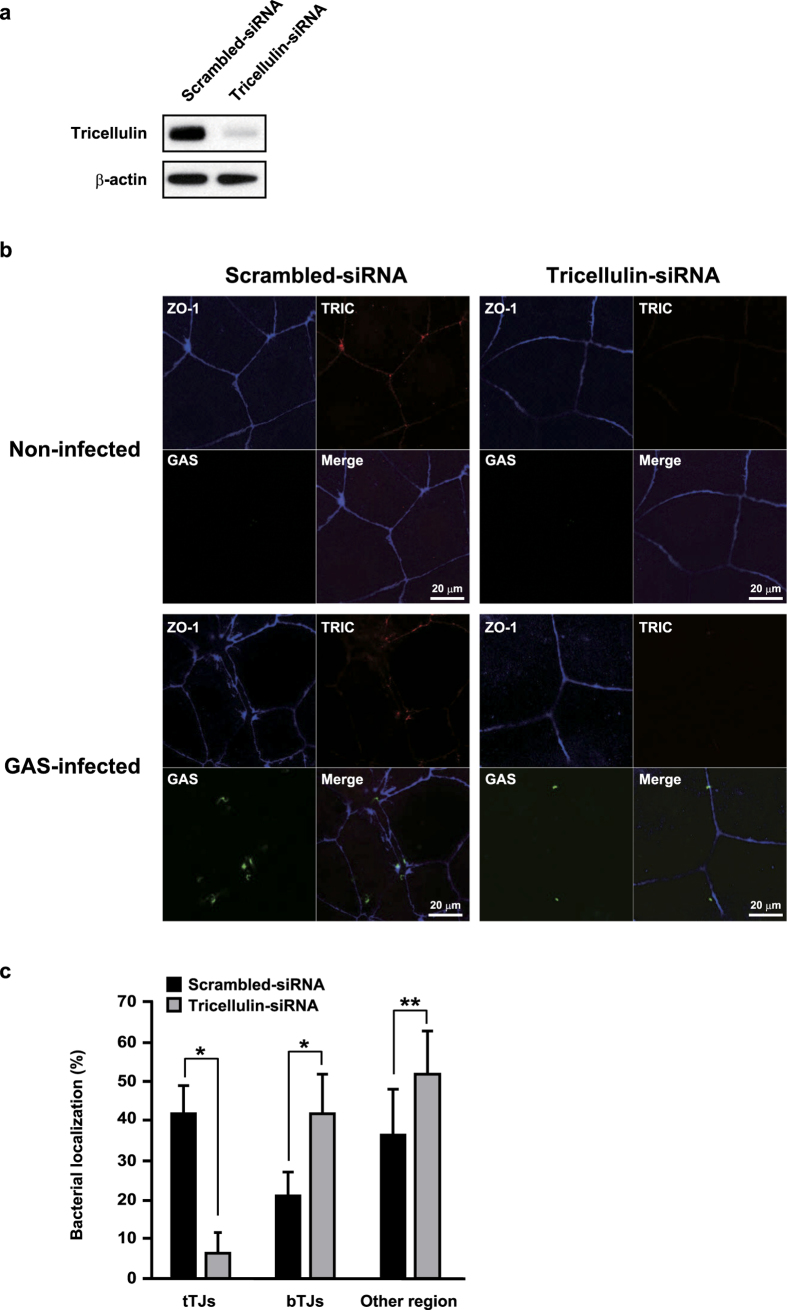
Tricellulin is a determinant for bacterial localization in tTJs. (**a**) Caco-2 cells were transfected with tricellulin-targeted siRNA. A scrambled siRNA was utilized as a negative control. At 72 h after transfection, whole cell lysates were subjected to Western blot analysis using anti-tricellulin antibody. β-actin served as a loading control. (**b**) Tricellulin knockdown or control cells were infected with EGFP-expressing GAS strain at an MOI of 10 for 2 h in the presence of 2 μM human PLG. ZO-1 and tricellulin were immunostained and are shown as blue and red images, respectively. GAS-infected cells were analyzed using a confocal laser-microscope. Data shown are representatives of at least three separate experiments. (**c**) Bacterial localization in tTJs, bTJs, or other regions was assessed as described in the Methods section. Data obtained from ten fields of view (x630) are presented as the mean ± S.D. *P < 0.01.

Tricellulin is a major component of tTJs and localized at an apical site in those junctions^8^, thus it is considered to be a putative determinant for PLG-mediated bacterial localization. To clarify the involvement of tricellulin in bacterial localization, the expression of tricellulin was silenced by siRNA. Western blot analysis showed that the level of tricellulin protein was effectively reduced by knockdown (Fig. 2a). Typical concentrated localization of tricellulin at tricellular contacts and co-localization of bacteria with tricellulin were observed in the scrambled siRNA-transfected control cells (Fig. 2b, Supplementary Fig. 2a). Interestingly, destruction of cellular contacts labeled with anti-ZO-1 antibody was found to be accelerated around GAS-associated sites in the control cells. On the other hand, with tricellulin knockdown, the fluorescent intensity for ZO-1 was decreased around the tricellular contacts. Although bacterial adherence to control cells and tricellulin-knockdown cells was nearly identical in regard to efficiency (Supplementary Fig. 2b), bacterial cells were found to be mainly distributed along the perimeter of paracellular junctions or in other regions. Quantitative analysis also showed that regardless of the presence of PLG, bacterial association with tTJs was remarkably inhibited by knockdown of tricellulin (Fig. 2c). Conversely, with tricellulin knockdown, the majority of bacterial cells were prone to localize with bTJs or in other regions. Together, these results suggest that the interaction between tricellulin and PLG is related to bacterial localization in tTJs, and results in destabilization of intercellular junctions.

### PLG binds to first extracellular loop of tricellulin

Human tricellulin is composed of cytoplasmic domains, four transmembrane domains, and two extracellular (EC) loops^8^. To test the interaction of tricellulin extracellular domains with PLG, we constructed His-tagged tricellulin EC1 and EC2 fragments, and then evaluated their ability to associate with PLG using surface plasmon resonance (SPR) measurement. Equilibrium dissociation constants for the binding of PLG to tricellulin proteins was calculated by association and dissociation curves applied to a 1:1 Langmuir binding model (Table 1). SPR analysis revealed that PLG binds to the tricellulin EC1 fragment with high affinity (*K*_D_ = 5.15 × 10^−8^ M), as compared with affinity for the EC2 fragment (*K*_D_ = 1.27 × 10^−6^ M) or mock protein (*K*_D_ = 8.38 × 10^−5^ M). PLG possesses five kringle domains that mediate the binding of PLG to lysine residues in PLG receptors^18^. Of note, the EC1 but not the EC2 loop of tricellulin contains lysine residues at positions 217 and 252, which were speculated to be plausible candidate PLG targets. To clarify whether the lysine residues of the EC1 loop contribute to binding, those lysine residues were substituted with alanine and subjected to SPR analysis. The K217A mutation (TRIC_EC1^K217A^) showed a slightly reduced affinity of EC1 for PLG (*K*_D_ = 1.83 × 10^−7^ M). Meanwhile, the ability of TRIC_EC1^K252A^ (*K*_D_ = 3.13 × 10^−6^ M) and TRIC_EC1^K217 + 252A^ (*K*_D_ = 1.03 × 10^−6^ M) to bind PLG was remarkably diminished, as compared with the wild type protein. These results indicate that PLG binds to the EC1 loop of tricellulin and the lysine residue at position 252 is relatively crucial for that interaction.

### PLG functions as a molecular bridge between SEN and tricellulin

**Figure 3:**
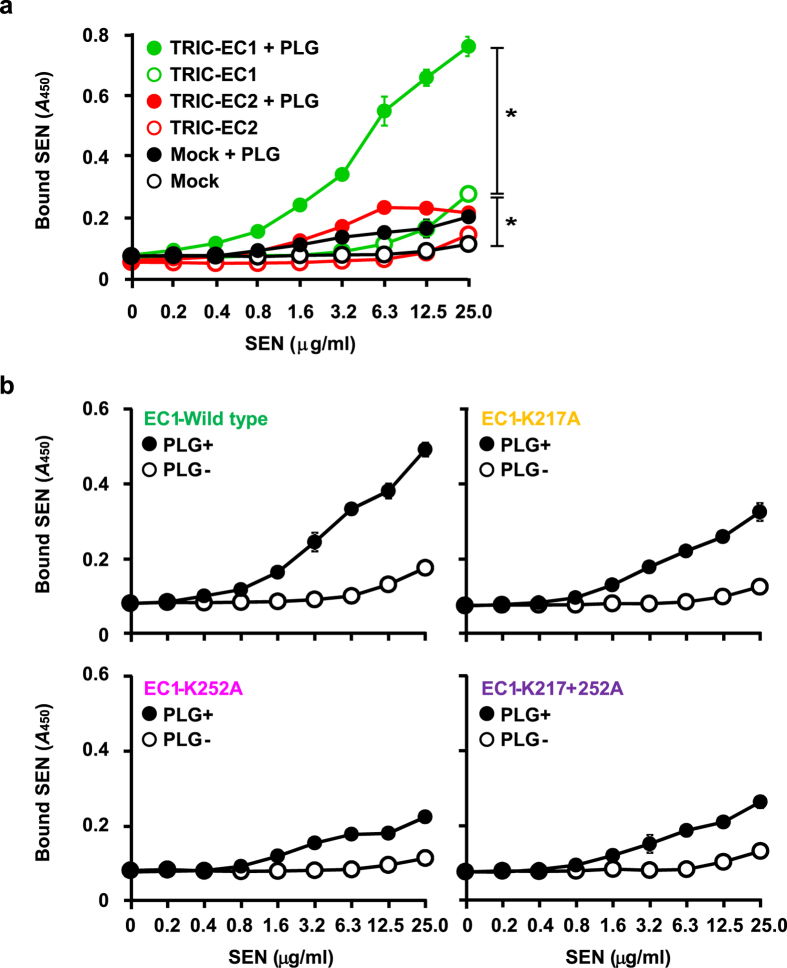
PLG functions as molecular bridge between SEN and tricellulin. TRIC proteins (**a**) or TRIC-EC1 variants (**b**) were immobilized on microtiter plates, then increasing amounts of SEN were added in the absence or presence of 1 μM human PLG. Bound SEN was detected using an anti-SEN antibody. All experiments were performed in sextuplet with three technical repeats. Data are shown as the mean ± S.D. of six wells from a representative experiment. *P < 0.01.

Since streptococcal surface enolase (SEN) is a major PLG receptor of GAS^19^,^20^, we speculated that GAS utilizes SEN-PLG interactions to associate with tricellulin. To examine our speculation, immobilized extracellular fragments of tricellulin were incubated with serially diluted SEN in the presence or absence of PLG, followed by detection with an anti-SEN antibody. While PLG had scant effects on binding of SEN to the EC2 fragment or mock protein, binding to the EC1 fragment was significantly accelerated in the presence of PLG (Fig. 3a). These data provide evidence that PLG acts as a molecular bridge between SEN and the EC1 domain of tricellulin. Additionally, the EC1 fragment had a slightly elevated affinity for SEN in the absence of PLG, as compared with the mock protein and EC2 fragment, which raised the possibility that the EC1 loop may directly bind SEN with low affinity. Next, the effects of lysine residues in the EC1 loop on PLG-mediated binding of SEN to tricellulin were investigated (Fig. 3b). There were no marked differences in binding activity of SEN to the EC1 variants observed in the absence of PLG. However, in agreement with the results of our SPR experiments, the capacity of SEN to bind TRIC_EC1^K252A^ and TRIC_EC1^K217 + 252A^ was greatly decreased in the presence of PLG, as compared with the wild type EC1 fragment. These results suggest that PLG mediates the association between SEN and tricellulin of tTJs.

### GAS translocates across epithelial barrier via interaction of SEN with human PLG

**Figure 4:**
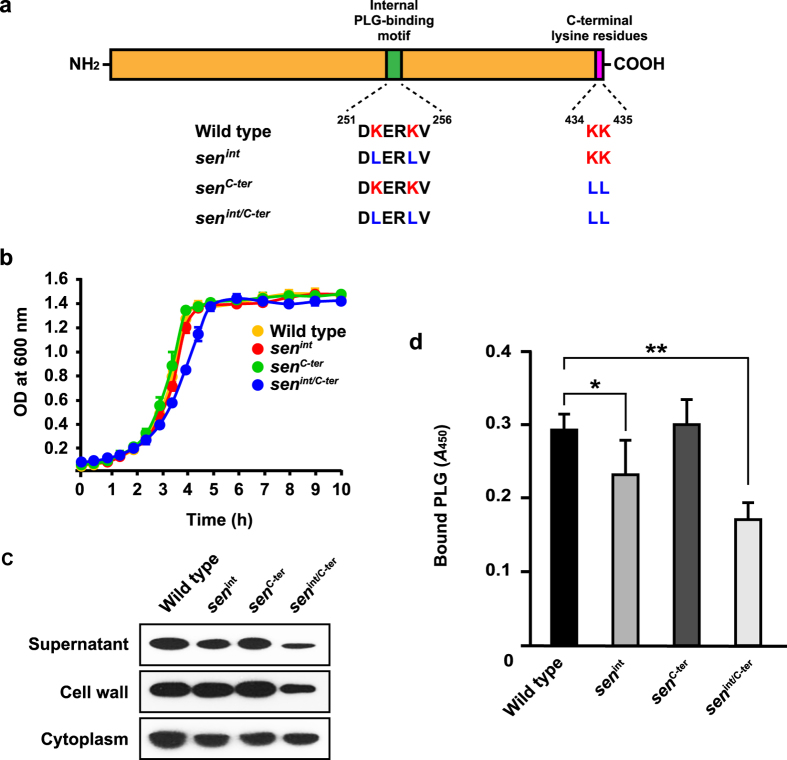
Introduction of sorting signal into SEN affects subcellular localization and bacterilal PLG binding. **(a)** Schematic diagram of SEN protein. Internal and C-terminus PLG-binding motifs are shown as green and pink images, respectively. The amino acid sequences of the PLG-binding motif in each strain are described below. (**b**) NIH35 and isogenic *sen* mutants were grown in THY broth. The culture densities were measured at 37 °C. (**c**) The strains were grown to an OD_600_ of 0.8 in THY broth, then each fraction was prepared, as described in Methods. (**d**) NIH35 and the isogenic *sen* mutants (OD_600_ = 0.6) were bound to microtiter plates, and bound cells were incubated with 1 μM human PLG. Cell-bound PLG was detected by ELISA using an anti-PLG antibody. Data are shown as the mean ± S.D. of six samples from a representative experiment. *P < 0.05; **P < 0.01.

Since the *sen* gene encoding SEN is essential for bacterial survival^21^, a knockout mutagenesis approach was not considered feasible for our study. The internal PLG-binding motif of SEN, which contains lysine residues at positions 252 and 255, and two C-terminus lysine residues at positions 434 and 435, are surface exposed and play essential roles in interaction with PLG^20^,^22^. Therefore, chromosomal site-directed mutagenesis of those SEN lysine residues in NIH35 was employed to verify whether an SEN-PLG interaction is required for bacterial translocation (Fig. 4a). Growth of the wild-type and mutant strains was nearly identical (Fig. 4b).

Prior to starting the translocation assay, the effect of mutagenesis on expression level and subcellular localization of SEN was examined (Fig. 4c). Substitution in the C-terminus lysine residues (*sen*^*C-ter*^) had no effect on SEN expression or localization of SEN. On the other hand, a slight reduction in SEN expression was detected in the supernatant and cell wall fractions obtained from the *sen*^*int*^ mutant. When both mutations were introduced (*sen*^*int/C-ter*^), the amount of secreted or exposed SEN was much less than that from the wild type. Also, mutagenesis of the surface-exposed lysine residues had no effect in respect to retention of SEN in the cytoplasm. Consequently, the lysine residues at positions 252 and 255 were considered to be more critical for secretion and exposure of SEN as compared to the C-terminus lysine residues.

Next, we examined the ability of the *sen* mutants to bind human PLG. Although it has been reported that PLG binding is attributed to the C-terminus lysine residues of SEN, the amount of bound PLG was not changed by replacement with leucine residues (Fig. 4d). In contrast, point mutations of either internal or all tested lysine residues inhibited the binding of PLG, indicating that internal lysine residues also contribute to PLG-binding of SEN.

**Figure 5:**
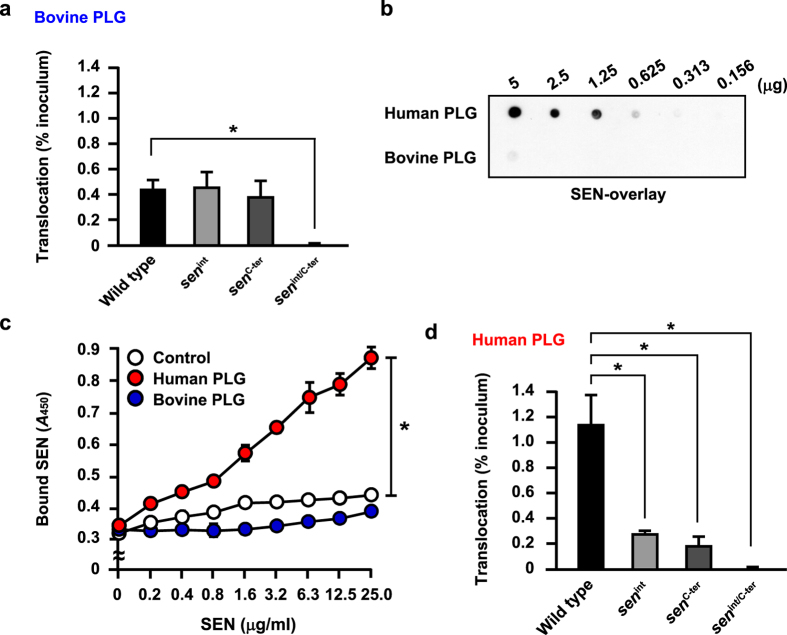
Bacterial translocation is mediated by interaction of SEN with human PLG. (**a**) Caco-2 cells were grown using a Millicell filter system, and then infected with the NIH35 strain or *sen* mutants at an MOI of 10 for 2 h. After removing non-adherent bacteria, the ability of the GAS strains to translocate across epithelial cells at 8 h after infection in the presence of bovine PLG was assessed by examining medium samples obtained from the lower chambers. Data are shown as the mean ± S.D. of six wells from a representative experiment. *P < 0.01. (**b**) Human and bovine PLG were immobilized on a nitrocellulose membrane after serial two-fold dilutions. After blocking of the membrane, 40 μg of SEN was overlaid and the binding signal was detected with an anti-SEN antibody. (**c**) TRIC-EC1 protein was immobilized on microtiter plates, and increasing amounts of SEN were reacted in the presence of 1 μM human PLG or 1 μM bovine PLG. Bound SEN was detected using an anti-SEN antibody. Data are shown as the mean ± S.D. from three independent experiments. *P < 0.01. (**d**) The effect of human PLG on bacterial translocation was analyzed as described in (**a**). Data are shown as the mean ± S.D. of six wells from a representative experiment. *P < 0.01.

We then performed a translocation assay using wild type and *sen* mutants cultured in MEM/20% FBS containing bovine PLG. Interestingly, the ability of GAS to translocate across the Caco-2 monolayer was not changed by mutations in either the internal or C-terminal lysine residues (Fig. 5a). In contrast, mutations in residues of both regions hampered bacterial translocation. In spite of the lower PLG-binding activity of *sen*^*int*^, translocation capacity was not decreased as compared with the wild type and *sen*^*C-ter*^. We considered that these conflicting results could be attributed to the interspecies variation of PLG. In fact, humans are the only known biological host of GAS. To investigate whether species specificity of PLG is related to SEN binding, dot spot overlay analyses using human and bovine PLG were employed. SEN bound strongly to human PLG in a concentration-dependent manner, whereas only slight binding to a high concentration of bovine PLG could be detected (Fig. 5b). ELISA data also demonstrated that the interaction of human PLG with SEN is more potent than that of bovine PLG (Fig. 5c). Based on these results, it is likely that SEN-mediated bacterial translocation is dependent on the species specificity of PLG. To verify that postulation, bovine PLG-depleted FBS was supplemented with 1 μM human PLG and subjected to a translocation assay. Mutagenesis in the surface-exposed lysine residues of SEN had no effect on capacity to adhere to the apical site of the Caco-2 monolayers (Supplementary Fig. 3). Interestingly, all mutants were partially impaired in regard to their ability to translocate across the epithelial barrier as compared with the wild type, indicating that SEN-PLG interaction and interspecies variations of PLG are important factors for bacterial penetration (Fig. 5d). However, the PLG-binding activity of *sen*^*C-ter*^ was not correlated with translocation rate. One possibility is that a PLG-independent mechanism by which C-terminus lysine residues in SEN directly interact with an uncharacterized host receptor is also involved in bacterial penetration. Moreover, mutations in both motifs abolished bacterial translocation regardless of the species specificity of PLG (Fig. 5a,d). Therefore, it seems likely that conformational changes caused by mutations can be attributed to the PLG-independent decrease in bacterial translocation.

### SEN is a bacterial determinant for GAS-induced cleavage of intercellular junctions

**Figure 6:**
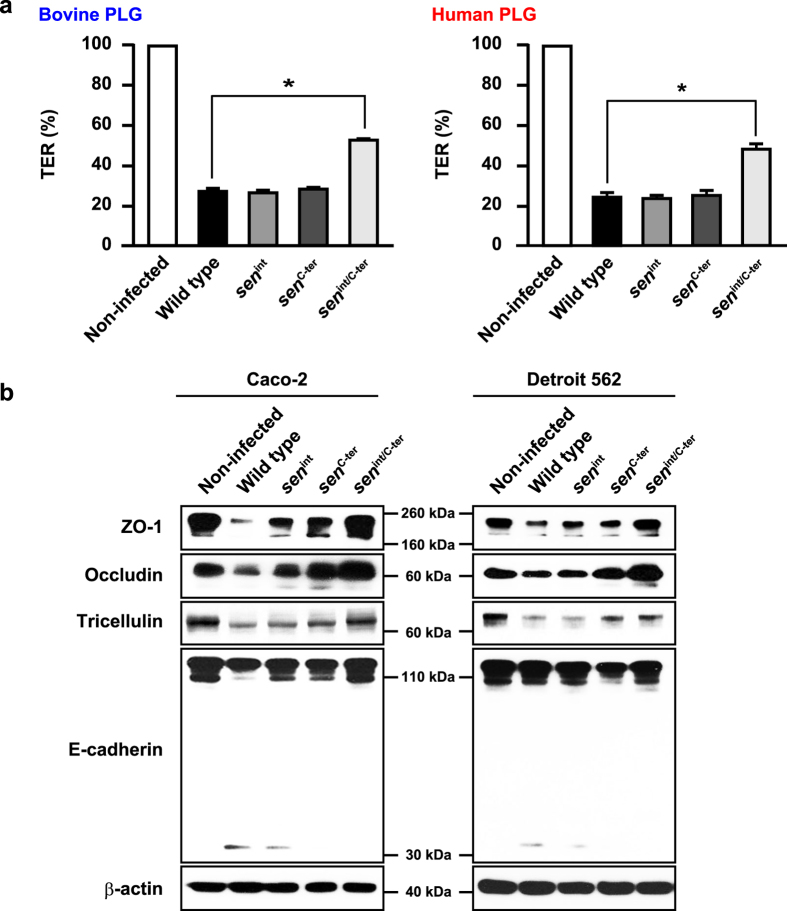
SEN is involved in GAS-induced disruption of intercellular junctions. **(a)** Effects of bovine or human PLG on reduction of TER in cells infected with GAS strains for 8 h. The TER value of the non-infected cells was set to 100%. All experiments were performed in sextuplet with three technical repeats. Data are shown as the mean ± S.D. of six wells from a representative experiment. *P < 0.01. **(b)** Caco-2 cells (left panel) or Detroit 562 cells (right panel) were infected with NIH35 or *sen* mutants at an MOI of 10 for 7 h in the presence of 1 μM human PLG. Cleavage of ZO-1, occludin, tricellulin, and E-cadherin was detected in whole cell lysates by Western blot analysis. β-actin served as a loading control.

We previously showed that several GAS strains efficiently translocate across epithelial monolayers, accompanied by a decrease in TER^10^. Consistent with previous reports, the values for TER in Caco-2 monolayers infected with the NIH35 strain were decreased by approximately 70% as compared with those in non-infected cells (Fig. 6a). Caco-2 monolayers infected with the *sen*^*int/C-ter*^ mutant exhibited a delayed decrease in TER value regardless of PLG species. Although the efficiency of bacterial translocation was decreased by mutagenesis in internal and C-terminus lysine residues of SEN, no significant difference in TER values between Caco-2 cells infected with the wild type strain or the *sen*^*int*^ or *sen*^*C-ter*^ mutants was observed. While TER is an index for TJ integrity, it is generally accepted that the relationship between the barrier properties of TJ strands and TER values is not linear^23^,^24^. Accordingly, destabilization of key components involved in maintenance of the epithelial barrier was examined. Marked cleavage of TJ proteins such as ZO-1, occludin, and tricellulin was detected in Caco-2 cells infected with the wild type strain, whereas that was prevented by mutations in surface-exposed lysine residues of SEN (Fig. 6b). A distinct cleavage product of E-cadherin with an apparent molecular mass of approximately 35 kDa was found in Caco-2 cells infected with the wild type or *sen*^*int*^ mutant strains. Furthermore, a 35-kDa fragment was detected using an antibody against the cytoplasmic domain of E-cadherin, suggesting that GAS induces cleavage of E-cadherin in the cytoplasmic region of infected cells. Meanwhile, no 35-kDa cleavage fragment was observed in Caco-2 cells infected with the *sen*^*C-ter*^ or the *sen*^*int/C-ter*^ mutant strains. Together, these results indicate that the C-terminus lysine residues of SEN are more important in GAS-induced destabilization of intercellular junctions. Similar observations were obtained using pharyngeal epithelial Detroit 562 cells. Our findings suggest that SEN-mediated barrier dysfunction of epithelial cells facilitates bacterial translocation via a paracellular route.

## Discussion

Microbial pathogens have evolved diverse strategies to establish colonization at preferred anatomical sites^25^,^26^. GAS is also able to acquire host PLG to the bacterial surface, which is known to play a crucial role in firm adherence to host epithelial cells^13^. PLG is abundant in saliva^27^,^28^, thus acquirement of PLG from saliva is important for GAS infection through the pharyngeal route. Several surface-exposed proteins have been identified as PLG receptors of GAS, whereas there is little information regarding host epithelial receptors interacting with GAS-bound PLG. In this study, we found that tricellulin is a key component for PLG-mediated bacterial association with tTJs. Since tricellulin plays a tightening role in establishment of the paracellular barrier^8^, GAS may exploit their PLG-binding property for tricellular localization followed by efficient bacterial penetration via a paracellular route.

Recruitment of PLG to the bacterial surface is mediated either by direct binding with specific surface proteins or indirectly via interactions with host plasma proteins such as fibrinogen^29^. GAS surface proteins involved in binding to PLG include glyceraldehyde-3-phosphate dehydrogenase [GAPDH; also known as streptococcal plasmin receptor, Plr, or streptococcal surface dehydrogenase (SDH)^30^,^31^], streptococcal surface enolase (SEN)^19^, plasminogen-binding group A streptococcal M protein (PAM)^32^, PAM-related protein (PrP)^33^, and extracellular protein factor (Epf)^34^. Although GAPDH was the first well-characterized protein shown to possess a direct ability to bind PLG or plasmin, it has been reported to bind PLG with low affinity^19^. PAM is found only in selected M types, such as M33, M41, M52, M53, and M56^32^,^35^. Meanwhile, SEN is ubiquitously expressed on the surface of nearly all M types and seems to be the most prominent receptor for PLG. Therefore, we hypothesized that PLG functions as a ‘molecular bridge’ between SEN and tricellulin. As expected, binding of SEN to the EC1 loop of tricellulin was significantly increased in the presence of PLG. These results suggest that the PLG-mediated interaction between SEN and tricellulin is a primary determinant for bacterial localization in tTJs. A slight affinity of SEN for the tricellulin EC1 fragment was also observed in the absence of PLG, implying that bacterial surface-exposed SEN is directly associated with tricellulin to a lesser extent.

A structure model of SEN based on the crystal structure of GAS and *S. pneumoniae* α-enolase indicates that SEN forms an octameric structure comprising a tetramer of dimer^22^,^36^,^37^. These models show that lysine residues of SEN at positions 252 and 255 within a unique lysine-rich PLG-binding motif are located in a surface-exposed loop at the edge of the toroidal octameric protein. The C-terminus lysine residues at positions 434 and 435 are also located adjacent to a dimer interface in the octamer, called ‘minor interface’. Although interactions between bacterial enolase and PLG have been characterized, there are conflicting reports regarding the mechanism. Consistent with eukaryotic enolase, the ability of SEN to bind PLG has been linked to C-terminus lysine residues^19^,^20^. On the other hand, recent ion mobility (IM) mass spectrometry (MS) analyses have shown that internal lysine residues also contribute to PLG binding^22^. Internal lysine residues in the closely related α-enolase of *S. pneumoniae* have also been reported to play crucial roles not only in the acquisition of PLG, but also in their virulence in a mouse infection model^38^. Recent structural and biophysical analyses of GAS SEN demonstrated that mutations in the minor inter-subunit interface induce destabilization of the octameric structure, thereby promoting the accessibility of PLG to its binding sites^37^. Since C-terminus lysine residues are located in the minor interface, we speculated that mutations in this region cause a conformational change of SEN, which might facilitate interaction of PLG with internal PLG-binding sites. However, the present results indicate that the internal PLG-binding motif plays a more important role in SEN-mediated bacterial binding to PLG, as compared to C-terminus lysine residues. In our experiments, the ability of GAS to bind with PLG was nearly completely abrogated by site-directed mutagenesis of both the internal and C-terminus lysine residues of SEN. Consequently, our findings imply that these two binding sites of SEN function in a synergistic manner to acquire PLG on the bacterial surface as part of the pathogenesis of GAS infections.

Confocal imaging of infected monolayers indicated that GAS preferentially translocate across the epithelial monolayer via the tTJs, accompanied by disruption of TJs. We found that SEN mediates GAS-induced cleavage of intercellular junctional proteins in the presence of human PLG. These results raised the possibility that streptokinase, a PLG activator secreted by GAS, is involved in that process. Mammalian PLG activators such as tissue-type PLG activator (tPA) and urokinase-type PLG activator (uPA) convert PLG to serine protease plasmin, which play central roles in tissue remodeling and blood clot removal following injury^39^. A wide variety of pathogens have been shown to hijack the PLG/plasmin system to promote tissue destruction and their own invasiveness^11^. Streptokinase also enables GAS to convert PLG to plasmin, which may be attributed to cleavage of the extracellular matrix and tissue barrier at primary sites of infection^12^. In our experiments with a serotype M28 strain, deletion of the *ska* gene encoding streptokinase had no effect on bacterial translocation across the epithelial monolayer in the presence of human PLG (data not shown). SpeB (streptococcal pyrogenic exotoxin B), a broad spectrum cysteine protease secreted by GAS, is known to effectively cleave streptokinase in the stationary phase^40^. Strain NIH35 used in this study possesses high SpeB activity, thus we were able to exclude the possibility that streptokinase is related to SEN-mediated bacterial translocation and destabilization of the intercellular junction in GAS-infected cells under the present experimental conditions.

Several GAS factors such as the hyaluronic acid capsule^41^, Streptolysin S (SLS)^10^ and SpeB^17^ have been identified as determinants for bacterial penetration through epithelial intercellular junctions. Herein, we examined whether substitution in the lysine residues of SEN could be associated with alterations in pathogenic phenotypes. We confirmed that capsule production, β-hemolytic phenotype, and SpeB-dependent proteolytic activity were not affected by mutagenesis (data not shown), indicating that the decreased translocation ability of the *sen* mutants was not attributed to effects of secondary mutations by site-directed mutagenesis. The molecular mechanism underlying SEN-mediated bacterial penetration remains elusive. Nevertheless, the present findings revealed that PLG acts as a linker molecule between surface-exposed SEN and tricellulin on the surface of host epithelium, facilitating bacterial localization to the tTJs. Therefore, SEN-mediated bacterial localization may facilitate the SLS- or SpeB-induced proteolytic action at tTJs.

GAS is a host-specific pathogen that naturally infects only humans. Although investigations using animal models have been restricted by host specificity, experimental evidence using transgenic mice expressing human PLG indicated that human PLG plays a critical role in systemic dissemination of GAS^42^. Streptokinase is known to be involved in determination of host species specificity for GAS infections. In this study, we demonstrated that the binding activity of SEN to PLG is dependent on the species-specificity of PLG, which has been shown to affect bacterial translocation across the epithelial barrier. Although further investigations such as *in vivo* animal studies are necessary for verification, the present results are the first to show that SEN is also a major determinant for host specific specificity in GAS infections.

Taken together, we found that PLG-mediated bacterial association with tricellulin is a crucial step for localization of GAS in tTJs. Our study is the first regarding the interaction of PLG with tricellulin. Furthermore, we report a novel biological aspect of SEN involved in mediating GAS transepithelial migration. These observations shed new light on the early stage of GAS infections and may provide important data for rational design of new drugs targeting that specific interaction.

## Methods

### Bacterial strains and culture conditions

GAS strain NIH35 (serotype M28) isolated from a patient with streptococcal toxic shock syndrome was kindly provided by Dr. H. Watanabe (National Institute of Infectious Diseases, Japan). Although we analyzed the chromosomal sequence of the *covRS* locus in the NIH35 strain, no mutation was detected. Among the clinical isolates examined, the NIH35 strain possesses relatively high levels of SLS and SpeB activities, whereas capsule production is low. NIH35 and isogenic mutant strains were cultured in Todd-Hewitt broth (Becton, Dickinson and Company; BD) supplemented with 0.2% yeast extract (BD) (THY medium) at 37 °C in an ambient atmosphere. *Escherichia coli* strain XL10-Gold (Stratagene) was used as a host for derivatives of pAT18^43^, pSET4s^44^, and pQE30 (Qiagen). *E. coli* strain BL21-CodonPlus (DE3)-RIPL (Agilent Technologies) served as a host for derivatives of pET-32a (+) (Novagen). All *E. coli* strains were cultured in Luria-Bertani (LB) (Sigma-Aldrich) at 37 °C with agitation. For selection and maintenance of mutant strains, antibiotics were added to the media at the following concentrations: ampicillin (Sigma-Aldrich), 100 μg ml^−1^ for *E. coli*; chloramphenicol (Sigma-Aldrich), 32 μg ml^−1^ for *E. coli*; erythromycin (Sigma-Aldrich), 150 μg ml^−1^ for *E. coli* and 1 μg ml^−1^ for GAS; spectinomycin (Sigma-Aldrich), 100 μg ml^−1^ for *E. coli* and GAS.

### Construction of GAS mutant strains

GAS mutant strains with amino acid substitutions in the SEN PLG-binding motif were constructed using the temperature-sensitive shuttle vector pSET4s, as previously reported^10^,^45^. Chromosomal point mutations in the *sen* gene were confirmed by sequence analysis of the locus encompassing the recombination sites. All primers used are listed in Supplementary Table 1.

To create EGFP-expressing GAS strains, a pAT18-EGFP vector was transformed into the GAS strains by electroporation. The transformants were grown in the presence of erythromycin.

### Cells

Caco-2 cells (Riken Cell Bank), a human colon carcinoma cell line, were cultured in minimum essential media (Life Technologies) supplemented with 20% heat-inactivated fetal bovine serum (SAFC Biosciences) (Matsumura CM). Detroit 562 cells (ATCC CCL-138), from a human pharyngeal cell line, were obtained from American Type Culture Collection and maintained in minimum essential medium-α (α-MEM, Wako) supplemented with 10% heat-inactivated Gibco fetal bovine serum (Life Technologies).

### Translocation assay

Bacterial translocation across epithelial cells was assessed, as previously described^10^. In brief, Caco-2 cells (4 × 10^5^ cells) were cultured on polycarbonate Millicell culture plate inserts (12-mm diameter, 3-μm pore size; Merck Millipore) at 37 °C under a 5% CO_2_ atmosphere. Transepithelial electrical resistance (TER) of the filter-grown monolayers was measured using a Milicell-ERS device (Merck Millipore) as an index for integrity of the TJs. Polarized monolayers exhibiting TER values of 450–500 Ω cm^2^ were used as an *in vitro* model of the epithelial barrier. The monolayers were infected with GAS strains at a multiple of infection (MOI) of 10. The ability of GAS strains to translocate across the epithelial barrier was assessed by quantitative cultures of media obtained from the lower chambers at 8 h after infection.

### Analysis of cleavage of junctional proteins

Whole cell lysates from GAS-infected epithelial cells were prepared as previously described^10^. Briefly, Caco-2 or Detroit 562 cells were infected with GAS strains at an MOI of 10. The infected cells were then lysed with Laemmli gel loading buffer containing 6% 2-mercaptoethanol at 7 h after infection. Cleavage of junctional proteins was detected by Western blot analysis with specific antibodies against ZO-1 (mouse mAb, Life Technologies), occludin (rabbit, pAb, Life Technologies), tricellulin (rabbit, mAb, Life Technologies), E-cadherin (mouse, mAb, Life Technologies) or β-actin (rabbit, pAb, Cell Signaling). Horseradish peroxidase (HRP)-conjugated antibodies against mouse or rabbit IgG (Cell Signaling) were used as the secondary antibodies. Immunoreactive bands were detected using Pierce Western blotting substrate (Thermo Scientific).

### RNA interference

Predesigned siRNAs against tricellulin (catalog number sc-92021) and negative controls (catalog number 12935-112) were purchased from Santa Cruz and Life Technologies, respectively. Caco-2 cells were transfected with three siRNA duplexes targeting different regions of the tricellulin gene at a final concentration of 150 nM. Transfection of siRNA was performed using an Amaxa Nucleofector according to the manufacturer’s instructions.

### Immunofluorescence confocal microscopy

Caco-2 cells were seeded at 2 × 10^5^ cells onto cover slides (13-mm diameter; Matsunami) pretreated with coating buffer containing 0.1% collagen (Type I from rat tail; Sigma-Aldrich) and 0.1% gelatin (from bovine bone; Wako), then cultured as described above. The cells were infected with EGFP-expressing GAS strains at an MOI of 10. At the end of the infection period, infected cells were fixed with 4% paraformaldehyde-PBS, followed by permeabilization with 0.2% Triton X-100. Following blocking with 5% bovine serum albumin-PBS, the cells were reacted with a primary antibody targeting human tricellulin (Rabbit, pAb, Merck Millipore) or ZO-1 (mouse, mAb, Life Technologies). After washing steps, the cells were incubated with Alexa Fluor 594-conjugated anti-rabbit IgG (Life Technologies) or Alexa Fluor 647-conjugated anti-mouse IgG (Life Technologies). Imaging was performed using a Zeiss LSM 510 confocal microscope system (version 3.2; Carl Zeiss) and analyzed with LSM 510 software. For assessment of bacterial localization in tTJs and bTJs, the numbers of tricellulin (red)-associated bacteria (green) colored in yellow and ZO-1 (blue)-associated bacteria colored in cyan were counted in 10 random fields of view. In this experiments, we regarded residual cell-associated bacteria (green) to be associated with other regions. Values presented show the percentage of bacteria among adhered bacteria associated with the respective regions.

### Preparation of recombinant proteins and antiserum

cDNA of Detroit 562 cells was prepared using Trizol and a PureLink RNA mini-kit (Life Technologies). cDNA fragments encoding the first extracellular loop (EC1) and the second loop (EC2) of tricellulin were amplified using specific primers (Supplementary Table 1). The fragments were cloned into a pET-32a (+) vector via *BamH*I and *Sal*I sites, and transformed into *E. coli* BL21-CodonPlus (DE3)-RIPL. A transformant carrying an empty plasmid (mock) was used as a negative control. For preparation of the EC1 variants, an overlapping PCR strategy was used. Primers were designed using the reported mRNA sequence of the Homo sapiens tricellulin isoform MARVELD2 (GenBank accession number DQ682656).

Recombinant SEN protein was hyper-expressed in *E. coli* XL10-Gold using a pQE30 vector. The N-terminal His-tagged SEN protein was purified using a QIAexpress protein purification system (Qiagen), as previously described^45^.

Mouse antiserum against SEN was raised by immunizing female BALB/c mice with the purified SEN protein, as previously reported^46^. Mouse immunization experiments for antiserum preparation were conducted under a protocol approved by the Animal Care and Use Committee of Osaka University Graduate School of Dentistry (Authorization number: 24-025-2).

### Bacterial cell fractionation

Culture supernatants, and cell wall/cytoplasm fractions of GAS strains were prepared as previously described^45^. Briefly, GAS strains were grown to the late exponential phase (OD_600_ = 0.8) in THY medium. Bacterial cells and culture supernatants were separated by centrifugation at 7000 × *g* for 5 min at 4 °C.

Total proteins in 7 ml of culture supernatant were precipitated with 20% trichloroacetic acid (Wako) at 4 °C overnight. The resulting precipitates were washed with ice-cold ethanol and resuspended in 15 μl of 1M Tris-HCl buffer (pH 8).

Bacterial cells harvested from the 7-ml culture samples were subjected to mutanolysin treatment in 300 μl of protoplasting buffer (0.1 M KPO_4_, pH 6.2, 40% sucrose, 10 mM MgCl_2_) containing 250 μg ml^−1^ of *N*-acetylmuramidase (Sigma-Aldrich) and Complete EDTA-free protease inhibitor (Roche Life Science) at 37 °C for 30 min. Following centrifugation, the resulting supernatants and pellets were obtained as cell wall and cytoplasm fractions, respectively. The pellets were resuspended in 1 ml of sterile water. All fractions were subjected to Western blotting analysis, as described above.

### Surface plasmon resonance analysis

Association and dissociation reactions of human PLG to tricellulin proteins were analyzed using a BIAcore optical biosensor (BIAcore X system, GE Healthcare Life Sciences), as previously described^47^. Briefly, tricellulin proteins (20 μg ml^−1^ in 10 mM sodium acetate, pH 5) were covalently immobilized on a CM5 sensor chip using an Amine coupling kit (GE Healthcare Life Sciences). Binding analyses were performed in HBS-P buffer (0.01 M HEPES, pH 7.4, 0.15 M NaCl, 0.005% surfactant P20; GE Healthcare Life Sciences) at 37 °C with a flow rate of 30 μl/min. Human PLG was used as an analyte at concentrations of 62.5, 125, 250, 500, or 1000 nM. Parameters of binding kinetics were analyzed using raw data from the BIAcore sensorgram suitable for analysis with the kinetic models included in BIA evaluation version 3.0.2 software (GE Healthcare Life Sciences). Data were fitted using a 1:1 Langmuir binding model.

### ELISA

PLG-dependent binding activity of SEN to tricellulin EC fragments was assessed by ELISA. Microtiter plates (96-well; Sumitomo Bakelite) were coated with tricellulin proteins (250 ng) in coating buffer (0.1 M Na_2_CO_3_, 0.1 M NaHCO_3_, pH 9.6) at 4 °C overnight. The plates were blocked with 10% Block Ace solution (Megmilk Snow Brand) at 4 °C overnight, then washed with PBS containing 0.2% Tween 20 (PBST). SEN was diluted with binding buffer (50 mM HEPES, pH 7.4, 150 mM NaCl, 2 mM CaCl_2_, 50 μg/ml BSA) and incubated with immobilized tricellulin proteins in the presence or absence of 1 μM PLG obtained from human plasma (Sigma-Aldrich) for 90 min at 37 °C. In other experiments, binding of SEN to bovine PLG was analyzed using PLG obtained from bovine plasma (Sigma-Aldrich). The plates were washed with PBST, then incubated with mouse antisera against SEN for 2 h at room temperature. Subsequently, an HRP-conjugated antibody against mouse IgG (Cell Signaling) was added to the plate and incubated for 2 h at room temperature. Following a washing step, the peroxidase substrate tetramethylbenzidine (Moss) was added to the plate. The reaction was stopped by addition of 0.5 N HCl and absorbance at 450 nm was measured using a Muktiskan FC microplate photometer (Thermo Scientific).

PLG binding to the GAS surface was also analyzed by ELISA. In brief, GAS strains were grown to the exponential phase (OD_600_ = 0.6) in THY medium and washed twice with PBS. Microtiter plates (96 wells; Sumitomo Bakelite) were coated with bacterial suspensions (1 × 10^7^ cfu) overnight at 4 °C. The plates were washed with PBST four times and blocked with 10% Block Ace solution, followed by incubation with PLG obtained from human plasma for 2 h at room temperature. Bound PLG was detected using an antibody against human PLG (mouse, mAb, R&D systems) and an HRP-conjugated antibody against mouse IgG, as described above.

### Protein dot spot analysis

PLG from human or bovine plasma was immobilized onto nitrocellulose membranes. After blocking with Block Ace solution at 4 °C overnight, the membranes were incubated with 40 μg of SEN at 4 °C for 4 h. After washing with PBST, the membrane was incubated with antisera against SEN for 1 h at room temperature. The membranes were then washed with PBST and incubated with an HRP-conjugated antibody against mouse IgG for 1 h at room temperature. Following washing, immunoreactive spots were detected using a Pierce Western blotting substrate.

### Statistical analysis

Statistical analysis was performed using Mann-Whitney’s *U* test. A confidence interval with a *P* value of < 0.05 was considered to be significant.

## Additional Information

**How to cite this article**: Sumitomo, T. *et al.* Group A *Streptococcus* exploits human plasminogen for bacterial translocation across epithelial barrier via tricellular tight junctions. *Sci. Rep.* **6**, 20069; doi: 10.1038/srep20069 (2016).

## Supplementary Material

Supplementary Information

## Figures and Tables

**Table 1:** Kinetic binding parameters for PLG to extracellular loops of tricellulin.

Ligand	*k*_a_ (M^−1^ s^−1^)	*k*_d_ (s^−1^)	*K*_D_ (M)
TRIC_EC1^WT^	1.39 × 10^8^	7.13	5.15 × 10^−8^
TRIC_EC1^K217A^	1.65 × 10^5^	2.98 × 10^−2^	1.83 × 10^−7^
TRIC_EC1^K252A^	5.89 × 10^3^	1.84 × 10^−2^	3.13 × 10^−6^
TRIC_EC1^K217+252A^	7.24 × 10^4^	7.42 × 10^−2^	1.03 × 10^−6^
TRIC_EC2	1.69 × 10^5^	2.14 × 10^−1^	1.27 × 10^−6^
Mock protein	5.02 × 10^3^	4.21 × 10^−1^	8.38 × 10^−5^
