# Erratum: Group A *Streptococcus* exploits human plasminogen for bacterial translocation across epithelial barrier via tricellular tight junctions

**DOI:** 10.1038/srep46388

**Published:** 2017-06-30

**Authors:** Tomoko Sumitomo, Masanobu Nakata, Miharu Higashino, Masaya Yamaguchi, Shigetada Kawabata

Scientific Reports 6: Article number: 20069; 10.1038/srep20069 published online: 01 29 2016; updated: 06 30 2017.

The original HTML version of this Article listed an incorrect volume number. This has now been corrected in the HTML version; the PDF version was correct at the time of publication.

