# Supplementary material for: Group A Streptococcus exploits human plasminogen for bacterial translocation across epithelial barrier via tricellular tight junctions
Source: Sci Rep. 2016 Jan 29;7:20069. doi: 10.1038/srep20069 (PMC4731814; doi:10.1038/srep20069)
Supplement: Supplementary Information [file srep20069-s1.pdf]

Supplementary information

Group A *Streptococcus* exploits human plasminogen for bacterial translocation across epithelial barrier via tricellular tight junctions

Tomoko Sumitomo, Masanobu Nakata, Miharu Higashino, Masaya Yamaguchi and Shigetada Kawabata

Sumitomo *et al.* Supplementary Table 1

Supplementary Table 1. Oligonucleotides used in this study.

Primer	Sequence (5'-3')	Purpose
sen252-255F1	CGGGATCCATGTCAATTATTACTGATGTATACGC	mutation of the *sen* gene
sen252-255R1	GTGTAGTCGTAAACAAGACGTTCCAAGTCGTAGAATTCTGATG	mutation of the *sen* gene
sen252-255F2	CATCAGAATTCTACGACTTGGAACGTCTTGTTTACGACTACAC	mutation of the *sen* gene
sen252-255R2	GCGTCGACGCTCAGGATAAATTAGCCCACAG	mutation of the *sen* gene
sen434-435F1	GCGTCGACTAAAGAACGTGGACTTGTTACAGC	mutation of the *sen* gene
sen434-435R1	CTGTCTTACCGTCTATAAGTTATAGAATGATTTGATACC	mutation of the *sen* gene
sen434-435F2	GGTATCAAATCATTCTATAACTTATAGACGGTAAGACAG	mutation of the *sen* gene
sen434-435R2	CGGGATCCACCAAACGTGGGAATCCTTATGC	mutation of the *sen* gene
rTRIC1F	GCGGATCCATTCATAAGGACAGTGAGTGGT	construction of recombinant TRIC
rTRIC1R	GCGTCGACCGGGGTCTTCGGGCCAGTGTAG	construction of recombinant TRIC
rTRIC1_K217A	CGGGATCCATTCATGCGGACAGTGAGTGGTA	construction of recombinant TRIC
rTRIC1_K252A	GCGTCGACCGGGGTCGCCGGGCCAGTGTA	construction of recombinant TRIC
rTRIC2F	GCGGATCCGTGAATGATACCAACCGCGGTG	construction of recombinant TRIC
rTRIC2R	GCGTCGACAATCTGTCCTCCTCTACGCGG	construction of recombinant TRIC
rSEN-F	CGGGATCCATGTCAATTATTACTGATGTATACGC	construction of recombinant SEN
rSEN-R	GCGTCGACCTATTTTTTTAAGTTATAGAATGATTTGA	construction of recombinant SEN

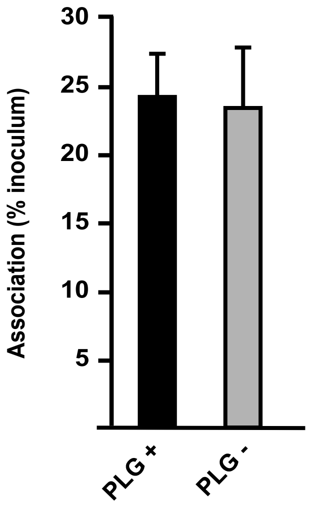

Supplementary Figure 1. Effect of PLG on bacterial adherence to epithelial cells.

Caco-2 cells grown on microplates were infected with NIH35 at an MOI of 10 in the presence or absence of 2 μM human PLG. At 2 h after infection, the cells were lysed and cell-associated GAS was recovered. Bacterial adherence rate was calculated as % inoculum. All experiments were performed in sextuplet with three technical repeats. Data are shown as the mean ± S.D. of six wells from a representative experiment.

a

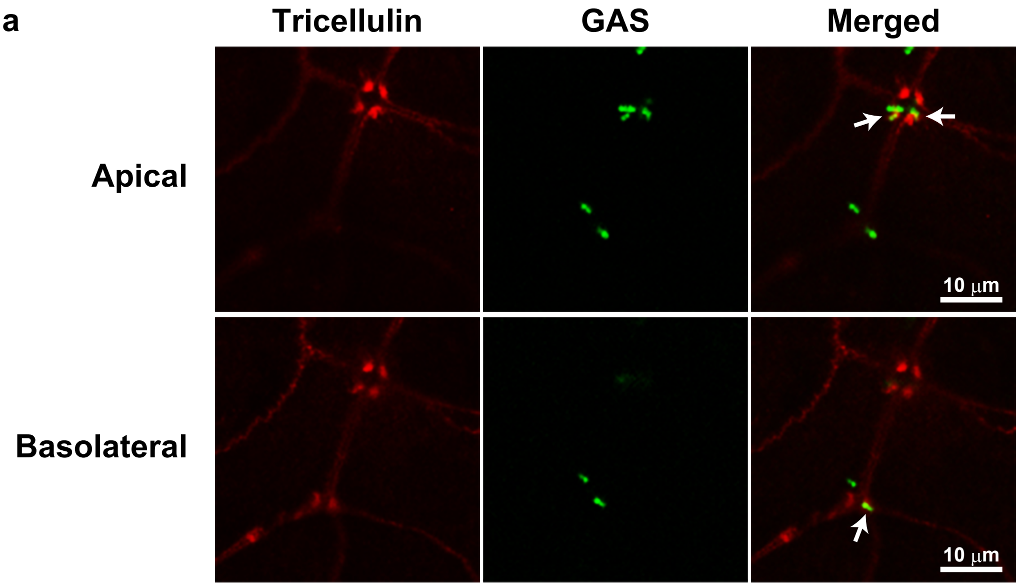

b

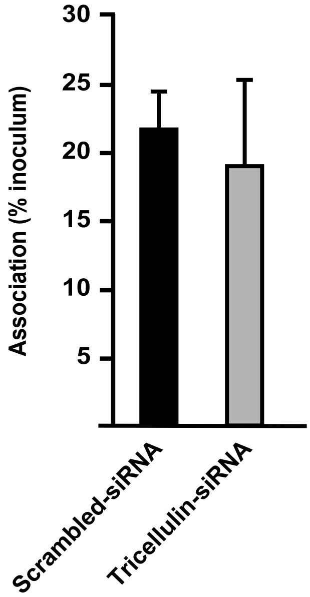

Supplementary Figure 2. Analysis of effects of tricellulin knockdown on other phenotypes.

(a) Representative images showing co-localization of GAS and tricellulin. Immunofluorescence staining of NIH35-infected Caco-2 cells was performed as described in Fig. 2. Upper and lower panels show contiguous z-stack images of infected cells transfected with control siRNA. White arrows indicate bacterial association with tricellulin. (b) Caco-2 cells were transfected with scrambled control oligonucleotides or tricellulin siRNA. At 72 h after transfection, cells were infected with NIH35 at an MOI of 10 in the presence of 2 μM human PLG. Bacterial adherence rate was calculated as % inoculum. Data are shown as the mean ± S.D. of six wells from a representative experiment.

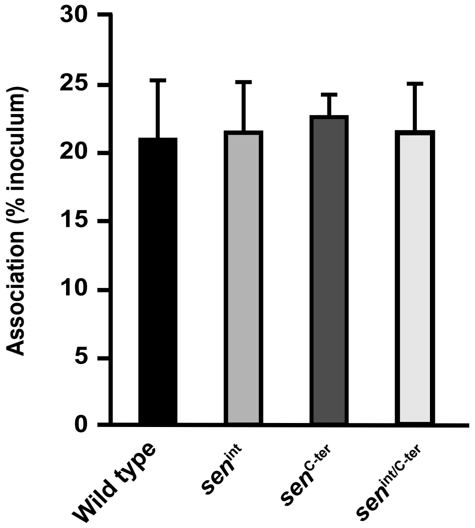

Supplementary Figure 3. Effect of the *sen* mutations on bacterial adherence to epithelial cells.

Caco-2 cells were grown on Millicell filters, then infected with NIH35 or *sen* mutants at an MOI of 10 in the presence of 2 μM human PLG. At 2 h after infection, the cells were lysed and cell-associated GAS was recovered. Bacterial adherence rate was calculated as % inoculum. All experiments were performed in sextuplet with three technical repeats. Data are shown as the mean ± S.D. of six wells from a representative experiment.
